# A Robust Structural PGN Model for Control of Cell-Cycle Progression Stabilized by Negative Feedbacks

**DOI:** 10.1155/2007/73109

**Published:** 2007-05-17

**Authors:** Nestor Walter Trepode, Hugo Aguirre Armelin, Michael Bittner, Junior Barrera, Marco Dimas Gubitoso, Ronaldo Fumio Hashimoto

**Affiliations:** 1 Institute of Mathematics and Statistics, University of São Paulo, Rua do Matao 1010, São Paulo, SP 05508-090, Brazil; 2 Institute of Chemistry, University of São Paulo, Avenue Professor Lineu Prestes 748, São Paulo, SP 05508-900, Brazil; 3 Translational Genomics Research Institute, 445 N. Fifth Street, Phoenix, AZ 85004, USA

## Abstract

The cell division cycle comprises a sequence of phenomena controlled by a stable and robust genetic network. We applied a probabilistic genetic network (PGN) to construct a hypothetical model with a dynamical behavior displaying the degree of robustness typical of the biological cell cycle. The structure of our PGN model was inspired in well-established biological facts such as the existence of integrator subsystems, negative and positive feedback loops, and redundant signaling pathways. Our model represents genes interactions as stochastic processes and presents strong robustness in the presence of moderate noise and parameters fluctuations. A recently published deterministic yeast cell-cycle model does not perform as well as our PGN model, even upon moderate noise conditions. In addition, self stimulatory mechanisms can give our PGN model the possibility of having a pacemaker activity similar to the observed in the oscillatory embryonic cell cycle.

## 

[1,2,3,4,5,6,7,8,9,10,11,12,13,14,15,16,17,18,19,20]
